# Cerebral amyloidoma mimicking intracranial tumor: a case report

**DOI:** 10.1186/1752-1947-4-308

**Published:** 2010-09-20

**Authors:** Danny Landau, Nicholas Avgeropoulos, Joe Ma

**Affiliations:** 1 MD Anderson Cancer Center Orlando, 1400 S Orange Boulevard, Orlando, Florida 32806, USA

## Abstract

**Introduction:**

Cerebral amyloidoma is an infrequently recognized condition that can be confused with a more malignant etiology. Few cases have been reported. We present a case report and a review of the literature.

**Case presentation:**

Our patient was a 64-year-old Caucasian man who was incidentally discovered to have a brain mass. He was found to have a cerebral amyloidoma.

**Conclusion:**

After discovery of the true etiology of his brain abnormality, it was determined that our patient had a more benign disease than was initially feared. Cases such as this demonstrate why consideration of this disorder is important.

## Introduction

Cerebral amyloidomas are rare entities infrequently described in the medical literature. Most commonly, they are noticed incidentally on brain scans. Frequently, they are confused with primary brain neoplasms. The clinical course tends to be benign, although long-term data is lacking.

## Case report

**Figure 1:**
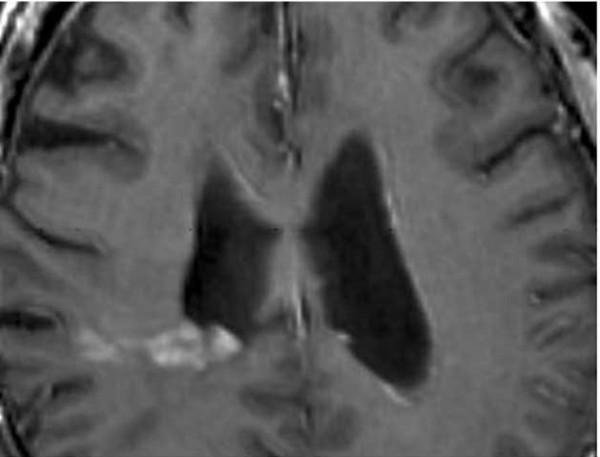
**Close up of an MRI showing enhancement along the right lateral ventricle**.

Our patient was a 64-year-old Caucasian man with a medical history of chronic obstructive pulmonary disease, coronary artery disease, and hyperlipidemia. He had been traveling, and experienced a transient loss of consciousness lasting a few seconds while in an airport abroad. He sought medical attention, and an initial computed tomography (CT) study of his head, without contrast, revealed a lobular hyperdensity in the right posterior periventricular distribution tracking to the adjacent parietal subcortical white matter. A follow-up magnetic resonance imaging (MRI) study revealed an infiltrating lesion extending from the right peritrigonal area across the corona radiata and into the subcortical white matter. The lesion showed increased T2-weighted and low T1-weighted signal intensity with post-gadolinium enhancement. Subcortical plaques were noted on fluid-attenuated inversion recovery (FLAIR) imaging (Figure 1). Our differential diagnosis included primary neoplasm, demyelinating or other inflammatory processes. A spectroscopy study revealed delayed perfusion without hyperperfusion within the enhanced area, suggestive of a small vessel process. Owing to the solitary space-occupying nature of the lesion, a surgical biopsy/excision was recommended and performed.

**Figure 2:**
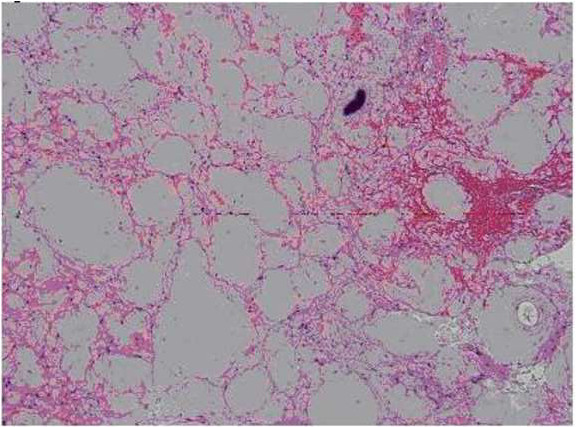
**Pathologic sections containing large confluent masses of pale eosinophilic deposits**.

**Figure 3:**
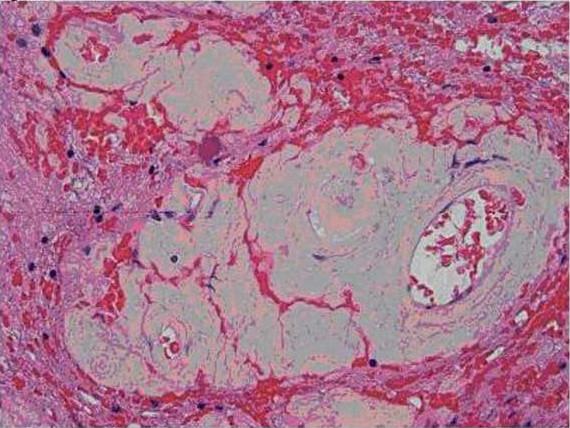
**Pathologic sections containing large confluent masses of pale eosinophilic deposits**.

**Figure 4:**
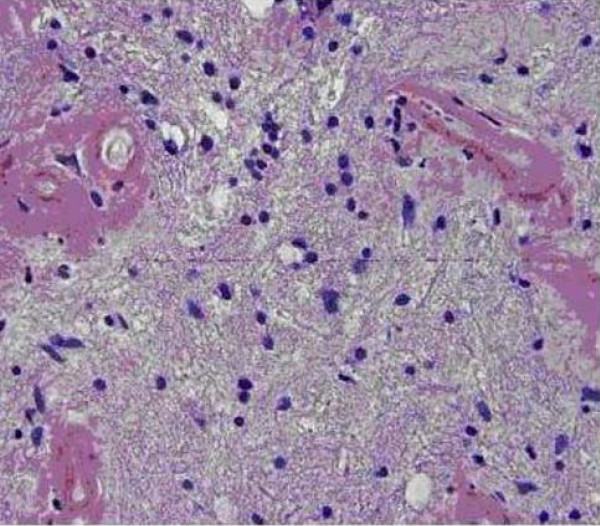
**An additional pathologic image showing congophilic staining**.

The surgically removed tissue consisted of abnormal and gliotic brain parenchyma containing large confluent masses of pale eosinophilic deposits (Figures 2 and 3) that were congophilic (Figure 4) with characteristic red-green birefringence under polarized light, consistent with amyloid. In areas where the amyloid deposits were not confluent, their perivascular distribution was apparent. Very focal calcification in the deposits was recognized, but no associated foreign body reaction was present. Immunohistochemical stains showed these deposits to have no immunoreactivity to antibodies against amyloid precursor protein (APP), kappa or lambda immunoglobulin light chain.

A staging evaluation was performed, including a CT scan of the chest, abdomen and pelvis, and an MRI scan of the spine to investigate for systemic findings of amyloidosis. The results of these tests were negative. To date, our patient remains well with no further episodes of seizure or neurological dysfunction. Follow-up MRI results have been negative to date. There are currently no plans for a repeat surgical procedure or repeat brain biopsies.

## Discussion

Amyloidosis is a heterogeneous group of diseases with complex pathogenesis and may take on many forms and manifest in various organ systems. The pathophysiology invariably results in extracellular deposition of insoluble proteins with β-pleated sheet as their secondary structure. It is the β-pleated sheet of amyloidogenic proteins that allows histochemical identification under light microscopy [1,2].

Amyloid deposition within the brain parenchyma can take on many forms, of which isolated amyloidomas are the least common [3,4]. More common forms of cerebral amyloidosis include senile plaques seen in ageing and Alzheimer disease (AD) and sporadic cerebral amyloid angiopathy (CAA), while hereditary cerebral amyloid angiopathy and cerebral autosomal dominant arteriopathy with subcortical infarcts and leukoencephatlopathy (CADASIL) are rare. The histopathology and distribution of cerebral amyloid found in our case are not consistent with any of these known entities [1,4-7].

A review of the literature reveals fewer than 30 reported cases [4,8]. Of these, the majority of presenting cases were initially thought to be primary intracerebral neoplasms. The average age of presentation is in the fourth decade, with a slight female predominance given the very finite number of cases available for review. As may be expected, clinical presentation is protean with seizure, headache, and cognitive decline reported. Cerebral white matter is the most commonly involved area, with lesions most often being supratentorial [8]. Typically, non-contrast CTs show hyperdensities that will enhance with contrast. MRI is more difficult to interpret due to variable results on imaging with some historically appearing hypointense, some isointense, and others hyperintense on T1 and T2 images [4].

The clinical course is thought to be benign, with no cases that were resected recurring. However, lesions that were biopsied without resection have shown growth [8]. Little is known about long-term effects on such patients as there are few published reports with data going beyond five years [8]. There are also limited data on surgical follow-up. The literature shows that most lesions were resected due to concerns of primary brain tumor. However, there have been no observed malignant transformations or other pathology related to amyloidomas that have been incompletely resected, therefore surgery is not necessary for the majority of patients with amyloidoma confirmed by biopsy [8]. There is no reported role for diffusion tensor imaging.

## Conclusions

Although rarely encountered, primary cerebral amyloidomas need to remain in the differential diagnosis of patients presenting with a solitary cerebral mass. While little is known regarding the long-term outcomes, after resection the disease does not appear to progress. With increased recognition, more data may become available on overall prognosis, but at this time it appears to represent a favorable clinical course.

## Consent

Written informed consent was obtained from the patient for publication of this case report and any accompanying images. A copy of the written consent is available for review by the journal's Editor-in-Chief.

## Competing interests

The authors declare that they have no competing interests.

## Authors' contributions

NA provided information on interpretation of scans and on patient outcomes. JM provided pathologic review of the brain biopsies. DL provided research into cerebral amyloidoma. All authors contributed equally to the writing of this report and agree with the final manuscript.
